# The Effectiveness of Biological Agents on Chronic Rhinosinusitis with Nasal Polyposis in Patients with Comorbid Asthma: A Multicenter Real-Life Study from Türkiye

**DOI:** 10.3390/medicina60030448

**Published:** 2024-03-08

**Authors:** Meryem Demir, Ceyda Tunakan Dalgic, Emine Nihal Mete Gokmen, Recep Savas, Suleyman Eroglu, Guzin Ozden, Cihan Orcen, Gulden Pacaci Cetin, Bahar Arslan, Ferda Bilgir, Gokten Bulut, Nurullah Yekta Akcam, Semiha Ozgul, Pamir Cerci, Raif Coskun, Sercan Gode, Insu Yilmaz, Aytul Zerrin Sin

**Affiliations:** 1 Division of Allergy and Immunology, Department of Internal Medicine, Ege University Faculty of Medicine, Izmir 35100, Türkiye; meryem.demir@ege.edu.tr (M.D.); ceyda.tunakan.dalgic@ege.edu.tr (C.T.D.); nihal.gomen@ege.edu.tr (E.N.M.G.); 2 Department of Radiology, Ege University Faculty of Medicine, Izmir 35100, Türkiye; recep.savas@ege.edu.tr; 3 Department of Otorhinolaryngology, Ege University Faculty of Medicine, Izmir 35100, Türkiye; suleyman.eroglu@ege.edu.tr (S.E.); sercan.gode@ege.edu.tr (S.G.); 4 Division of Allergy and Immunology, Department of Internal Medicine, Adana City Hospital, Adana 01230, Türkiye; guzin.ozden1@sbu.edu.tr; 5 Division of Allergy and Immunology, University of Health Sciences, Derince Training and Research Hospital, Kocaeli 41900, Türkiye; c_orcen@hotmail.com; 6 Division of Allergy and Immunology, Erciyes University Faculty of Medicine, Kayseri 38039, Türkiye; guldenpacacicetin@erciyes.edu.tr (G.P.C.); bahararslan@erciyes.edu.tr (B.A.); insu@erciyes.edu.tr (I.Y.); 7 Department of Allergy and Immunology, Ataturk Training and Research Hospital, Katip Celebi University, Izmir 35360, Türkiye; ferda.bilgir@saglik.gov.tr; 8 Division of Allergy and Immunology, Ataturk City Hospital, Balikesir 10100, Türkiye; gokten.bulut@saglik.gov.tr; 9 Division of Allergy and Immunology, Mersin City Hospital, Mersin 33230, Türkiye; n.akcam@saglik.gov.tr; 10 Department of Biostatistics and Medical Informatics, Ege University Faculty of Medicine, Izmir 35100, Türkiye; semiha.ozgul@ege.edu.tr; 11 Division of Allergy and Immunology, Eskisehir City Hospital, Eskisehir 26080, Türkiye; pamir.cerci@saglik.gov.tr; 12 Division of Allergy and Immunology, Cemil Tascioglu City Hospital, Istanbul 34384, Türkiye; raifcoskun1985@yahoo.com

**Keywords:** omalizumab, mepolizumab, nasal polyposis, CRSwNP, asthma, biological agents

## Abstract

*Background and Objectives*: Real-life data on the efficacy of biologic agents (BAs) on asthma-comorbid CRSwNP are needed. Our primary goal is to investigate the effects of BAs on CRSwNP symptoms, as well as endoscopic and tomography scores. Our secondary goal is to show a reduction in the frequency of acute sinusitis exacerbations and the need for surgery. *Materials and Methods*: We conducted a multicenter, retrospective, real-life study. We screened the patients with asthma-comorbid CRSwNP treated with omalizumab or mepolizumab. A total of 69 patients (40 F/29 M; omalizumab n = 55, mepolizumab n = 14) were enrolled. We compared the visual analog scale (VAS), sinonasal outcome test-22 (SNOT-22), nasal congestion score (NCS), Lund–Mackay computed tomography score (LMS), and total endoscopic polyp scores (TPS) before and after BAs. We evaluated the endoscopic sinus surgery (ESS) and acute exacerbations of chronic rhinosinusitis (AECRS) frequencies separately, according to the BAs. *Results:* The overall median (min–max) age was 43 (21–69) years. The median (min–max) of biologic therapy duration was 35 (4–113) months for omalizumab and 13.5 (6–32) for mepolizumab. Significant improvements were seen in VAS, SNOT-22, and NCS with omalizumab and mepolizumab. A significant decrease was observed in TPS with omalizumab [95% CI: 0–4] (*p* < 0.001), but not with mepolizumab [95% CI: −0.5–2] (*p* = 0.335). The frequency of ESS and AECRS were significantly reduced with omalizumab [95% CI: 2–3] (*p* < 0.001) and [95% CI: 2–5] (*p* < 0.001); and mepolizumab [95% CI: 0–2] (*p* = 0.002) and [95% CI: 2–8.5] (*p* < 0.001), respectively. There was no significant difference in LMS with either of the BAs. *Conclusions:* Omalizumab and mepolizumab can provide a significant improvement in the sinonasal symptom scores. BAs are promising agents for CRSwNP patients with frequent exacerbations and multiple surgeries.

## 1. Introduction

Chronic rhinosinusitis (CRS) is an inflammation of the nasal cavity and paranasal sinuses defined as the presence of ≥2 symptoms, consisting of nasal obstruction/congestion, nasal discharge, facial pressure, and the reduction/loss of smell for ≥12 weeks [1]. Primary CRS been traditionally classified as CRS with nasal polyps (CRSwNP) and without nasal polyps (CRSsNP) [1].

The following four overlapping classification schemes have emerged to define the CRSwNP endotypes: (1) the immunoglobulin (Ig)E-based approach, (2) the type 2 cytokine-based approach, (3) the eosinophil-based approach, and (4) the cysteinyl-based approach [2].

Patients with CRS can be classified pathophysiologically into three endotypes as type 1, type 2, or type 3 [3]. Bilateral diffuse primary CRS may be phenotypically present as CRSwNP/eosinophilic CRS (eCRS), non-eosinophilic CRS (neCRS), and allergic fungal sinusitis (AFS) [4]. The phenotypes do not provide a full insight into all of the underlying mechanisms; however, CRSwNP associated with asthma is generally a type 2 endotype and recurrent form [1,5].

The standard treatment for CRSwNP consists of medical management with nasal saline irrigations, intranasal corticosteroids, short-term systemic corticosteroids, and antibiotics. Endoscopic sinus surgery (ESS) may be needed in patients who are unresponsive to medical treatments [6]. CRSwNP patients with recurrent systemic corticosteroid usage and repeated sinus surgeries generally have high symptomatology and a significantly impaired quality of life (QoL) [5,7,8,9].

Asthma has several phenotypes, and some of these require advanced therapies [10]. Targeted biological agents (BAs) have been successfully used in severe, uncontrolled type 2 asthma phenotypes [10,11].

The rate of CRSwNP in severe asthma is approximately ten-fold that of the normal population [12,13]. The combination of asthma and CRSwNP causes frequent asthma exacerbations, difficulties in the treatment, and a significant worsening of QoL [6,14].

Mainly, the treatments for CRSwNP of type 2 or mixed endotypes are challenging, due to resistance to medicines, and have low ESS success. These patients may require repetitive surgeries, systemic steroids, or antibiotics, with frequent acute sinusitis exacerbations (AECRS).

DeConde et al. showed that patients who had ESS due to nasal polyposis had recurrence rates of 35%, 38%, and 40% at the 6th, 12th, and 18th months, respectively [15]. There are other publications showing recurrence rates of up to 50–60%, varying depending on the surgical procedure, the severity of nasal polyposis, and the concomitant medical treatment [16,17].

BAs have been successfully used for severe asthma for many years, and their benefits in CRSwNP treatment have begun to be defined recently [18,19].

The U.S. Food and Drug Administration approved dupilumab, anti-IL-4/IL-13; 2019, omalizumab, anti-IgE; 2020, and mepolizumab, anti-IL-5; 2021 to treat adults with CRSwNP [20]. Since there is no access to BAs within the scope of public health insurance for CRSwNP in Türkiye, many patients are deprived of these agents.

In this real-life study, we aimed to determine the effectiveness of omalizumab and mepolizumab treatment on comorbid CRSwNP in patients who were treated for severe asthma. Since Dupilumab has not yet been used for asthma in Türkiye, it could not be included in this study. Our primary goal is to define the efficacy of BAs on nasal polyposis recurrence. Our secondary goal is to determine the improvement of sinonasal symptom scores and endoscopic and radiological findings after BAs.

## 2. Materials and Methods

### 2.1. Study Design and Patient Population

We conducted a multicenter, retrospective, observational, real-life study in the Division of Allergy and Immunology, Department of Internal Medicine of Ege University, Izmir, Türkiye. Nine different allergy and immunology centers participated in this study. Before the study, central and local ethics committee approval was obtained (Approval numbers: 20-12T/32, 21-4T/71) and written informed consent was retrieved.

All asthma-comorbid CRSwNP patients who had been receiving BAs between January 2010 and December 2021 were screened. Patients aged between 18 and 65 years, with a diagnosis of asthma defined according to the Global Initiative for Asthma (GINA) criteria [10], with a concomitant endoscopic- or paranasal-CT-confirmed CRSwNP diagnosis, receiving omalizumab or mepolizumab treatment for ≥4 months, and those who agreed to participate in this study were considered eligible for inclusion. The dose (in milligrams) and dosing frequency (every 2–4 weeks) of omalizumab were based on total serum IgE levels (IU/mL) and body weight (in kilograms), and standard 100 milligrams every month for mepolizumab subcutaneously, as mentioned commercially.

The patients with active malignancy, severe active autoimmune disease, and non-polyp chronic sinonasal and non-asthmatic chronic lung disease; those using of BAs for <4 months; and those who did not want to be included in this study were not included.

The baseline eosinophil and total IgE values of the patients before BA are presented. Safety is not the primary focus of this study, and the reported side effects were recorded.

The patients were analyzed in 2 separate subgroups as those using omalizumab or mepolizumab. The post-treatment patient-reported outcomes and endoscopic polyp scores were evaluated at the patients’ last visit. This evaluation interval is equivalent to BA treatment durations and is heterogeneous. Since the patients included in our study took BAs for a very long time (min–max: 4–113 months), all medical records could not be accessed for some patients.

### 2.2. Patient-Reported Outcomes

The patient-reported outcomes included the ‘Visual Analogue Scale (VAS)’, ‘Nasal Congestion Score (NCS)’, and ‘Sinonasal Outcome Test (SNOT-22)’ for the sinonasal symptoms. Because of the known efficacy of BAs in asthma treatment, we focused on the real-life effectiveness of BAs on CRSwNP specifically. We assessed the scores before and after BA administration. The comparison of the patient-reported outcome (PRO) intervals was equal to the BA treatment durations. 

Post-nasal drip, runny nose, nasal obstruction, and loss of smell were scored with VAS from 1 to 10 points [21]. The value of 0 points means no complaint, while 10 points means the worst complaints.

For NCS assessment, the patients were asked to assess their degree of nasal congestion on a 0- to 3-point scale [22].

SNOT-22 is a valuable health-related quality of life questionnaire that is widely used in chronic rhinosinusitis. We used the Turkish validated SNOT-22 to reflect the situation of the patient’s baseline and post-treatment outcomes regarding physical, functional, emotional, and social conditions [23].

Acute exacerbation of chronic sinusitis was defined as the worsening of symptom severity with the return to baseline symptom severity, requiring corticosteroids and/or antibiotics. The annual frequency of AERCS before and after BA administration was questioned, and was confirmed from national health system data. 

### 2.3. Lund–Mackay Score

The baseline and post-treatment paranasal computed tomography (CT) scans were evaluated by using the Lund–Mackay score (LMS), a quantitative comparative score, performed by the same experienced radiologist in the executive center. Each sinus group was graded between 0 and 2 (0: no abnormality; 1: partial opacification; 2: total opacification). The osteomeatal complex was scored as “0” (not obstructed) or “2” (obstructed). The right and left sinuses were scored between 0 and 12, and the total score was obtained between 0 and 24) [24,25].

### 2.4. Total Endoscopic Nasal Polyp Score 

The initial and post-treatment endoscopic total nasal polyp scores (TPS) performed by their otolaryngologists were recorded. Each nostril was scored on a scale of 0 to 4, with the total score being the sum of the left and right nostril scores (0–8). The score was revealed as follows: 0: no nasal polyps; 1: small nasal polyps in the middle meatus not reaching below the inferior border of the middle turbinate: 2: nasal polyps reaching below the lower border of the middle turbinate; 3: large nasal polyps reaching the lower border of the inferior turbinate or polyps medial to the middle turbinate; and 4: large nasal polyps causing complete obstruction of the inferior nasal cavity [22].

### 2.5. Statistical Analysis

The categorical baseline characteristics were summarized with frequencies and percentages, while numerical ones were summarized with mean and standard deviation (SD). The primary outcomes were presented with median and its exact 95% confidence intervals (CIs) [26]. The amount of change in primary outcomes over the time course were summarized with median difference (MD) and its 95% CIs (95% CIs were calculated according to the bootstrap percentile method, n = 1000). The change in clinical scores before and after biologics were analyzed with the nonparametric Brunner–Langer model (F1-LD-F1). The effect estimate that was exactly consistent with the Brunner–Langer model was the relative treatment effect estimator (calculated as (rank means -1/2)/N); however, for convenience, the median was chosen, as it provides parallel interpretations. The reason why the interpretation of the confidence intervals of the MD of some variables and the relevant *p* value interpretation did not coincide was due to this choice.

Box plots were used for graphical representations. In the boxplots, the horizontal lines of the rectangles from bottom to top show the 1st quartile, the 2nd quartile (median), and the 3rd quartile, respectively. The vertical lines extend from the boxplot as 1.5 times the interquartile range. The points outside these lines indicate potential outliers. 

The statistical significance was assessed at *p* < 0.05, and all statistical analyses were performed using R software (R software, version 4.0.5, package: arsenal, R Foundation for Statistical Computing, Vienna, Austria; http://rproject.org, accessed on 1 January 2021).

## 3. Results

### 3.1. Patients’ Demographics

In total, we included 69 patients (n = 69, 40 females, 29 male) in this study. Fifty-five patients (n = 55) were receiving omalizumab and fourteen patients (n = 14) were receiving mepolizumab for asthma-comorbid CRSwNP. The overall median (min–max) age was 43 (21–69) years. 

The median (min–max) of biologic therapy duration was 35 (4–113) months for omalizumab and 13.5 (6–32) for mepolizumab.

While 61 (88.4%) of the 69 patients with severe asthma were receiving BAs, 8 (11.5%, omalizumab: 7, mepolizumab: 1) with mild/moderate asthma-comorbid isolated nasal polyposis were receiving off-label, due to the side effects of steroids. Off-label use approvals were received from the Turkish Pharmaceutical and Medical Device Agency.

The baseline median (IQR) eosinophil values of the patients were significantly higher in the mepolizumab group (eosinophil count, cells/µL, 1175 (406–2400) vs. 630 (0–3800)), (*p* = 0.003), while the baseline median (IQR) total IgE level was quantitatively higher in the omalizumab group (291 (21–2333) vs. 280.5 (44–738)); however, there was no statistically significant difference. House dust mite sensitivity was higher in the omalizumab group (85.5% vs. 14.3%, *p* < 0.001) (Table 1).

### 3.2. Patient-Reported Outcomes

The median (IQR) pre- and post-treatment PRO evaluation intervals were 35 (4–113) months for omalizumab and 13.5 (6–32) months for mepolizumab.

#### 3.2.1. Visual Analogue Scale (VAS)

Significant improvements in postnasal drip, runny nose, and nasal congestion VAS scores were observed with both BAs.

**Figure 1:**
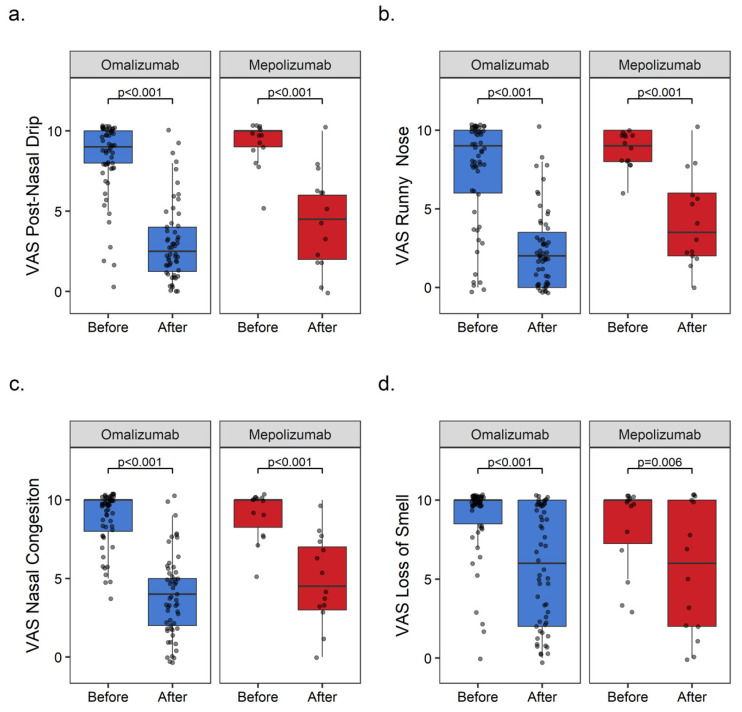
Boxplots of improvements of VAS with omalizumab and mepolizumab. (**a**) VAS-post-nasal-drip, (**b**) VAS-runny-nose, (**c**) VAS-nasal-congestion, and (**d**) VAS-loss-of-smell.

The median VAS-postnasal-drip reduced from 9 [95% CI: 8–10] to 2.5 [95% CI: 2–3] (*p* < 0.001) with omalizumab and from 10 [95% CI: 8–10] to 4.5 [95% CI: 2–8] (*p* < 0.001) with mepolizumab. The reductions in the median VAS-runny-nose score were observed as 9 [95% CI: 8–10] to 2 [95% CI: 1–3] (*p* < 0.001) with omalizumab and 9 [95% CI: 8–10] to 3.5 [95% CI: 2–8] (*p* < 0.001) with mepolizumab. The median VAS-nasal-congestion reduced from 10 [95% CI: 9–10] to 4 [95% CI: 3–5] (*p* < 0.001) with omalizumab and from 10 [95% CI: 8–10] to 4.5 [95% CI: 3–8] (*p* < 0.001) with mepolizumab. There was a significant improvement in the loss of smell in the omalizumab group, but not in the mepolizumab group. The mean differences (MD) in VAS-loss-of-smell were −4 [95% CI: −1, −6] (*p* < 0.001) with omalizumab and −4 [95% CI: 1, −8] (*p* = 0.006) with mepolizumab (Figure 1).

#### 3.2.2. Nasal Congestion Score (NCS)

**Figure 2:**
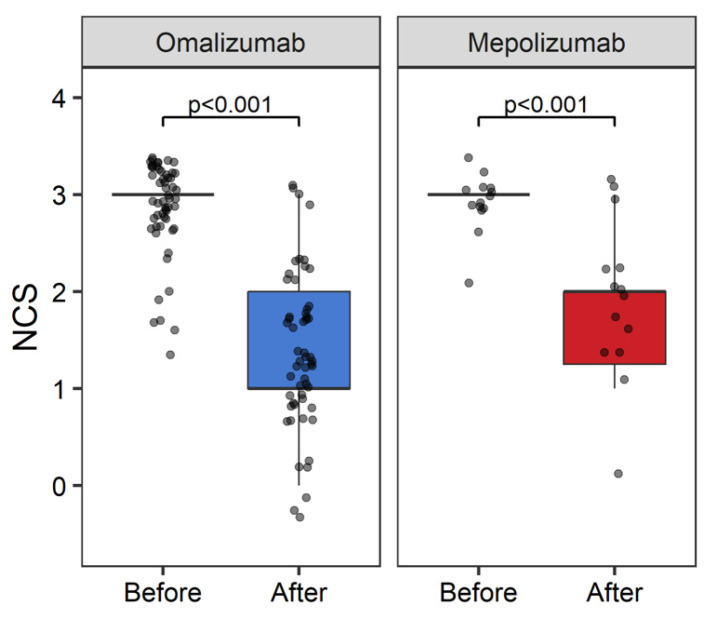
Improvement of nasal congestion score after biological agents.

We observed that there were significant improvements in the nasal congestion score with both BA groups after therapy compared to before. Respectively, the median NCS was reduced from 3 [95% CI: 3–3] to 1 [95% CI: 1–2] (*p* < 0.001) in the omalizumab group, and from 3 [95% CI: 3–3] to 2 [95% CI: 1–3] (*p* < 0.001) in the mepolizumab group (Figure 2).

#### 3.2.3. Sinonasal Outcome Test-22 (SNOT-22)

**Figure 3:**
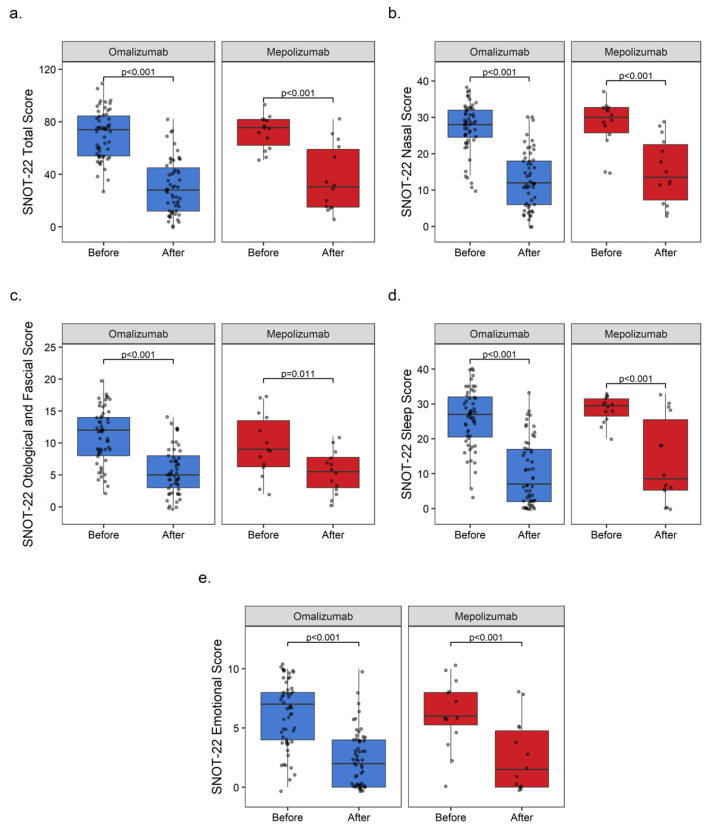
Boxplots of SNOT-22 total and subgroup scores of omalizumab and mepolizumab. (**a**) SNOT-22 total score, (**b**) SNOT-22 nasal score, (**c**) SNOT-22 otological/fascial score, (**d**) SNOT-22 sleep score, and (**e**) SNOT-22 emotional score.

Considering the SNOT-22 nasal, otological/facial, sleep, and emotional subgroup analyses and SNOT-22 total values, significant improvements were recorded in all of the parameters after treatment with omalizumab and mepolizumab. The MD reductions in the SNOT-22 total score were −46 [95% CI: −30, −54] (*p* < 0.001) with omalizumab and −45 [95% CI: −14.5, −63.5] (*p* < 0.001) with mepolizumab. The MD reductions in the SNOT-22 nasal symptoms were −16 [95% CI: −12, −19] (*p* < 0.001) and −16.5 [95% CI: −6.5, −23.5] (*p* < 0.001), the SNOT-22 otological and fascial symptoms were −7 [95% CI: −3, −8] (*p* < 0.001) and −3.5 [95% CI: 0.5, −9] (*p* = 0.011), the SNOT-22 sleep symptoms were −20 [95% CI: −13, −23] (*p* < 0.001) and −21 [95% CI: −2.5, −25.5] (*p* < 0.001), and the SNOT-22 emotional symptoms were −5 [95% CI: −3, −6] (*p* < 0.001) and −4.5 [95% CI: −1.5, −7.5] (*p* < 0.001) with omalizumab and mepolizumab, respectively (Figure 3).

### 3.3. Endoscopic Total Nasal Polyp Scores 

**Figure 4:**
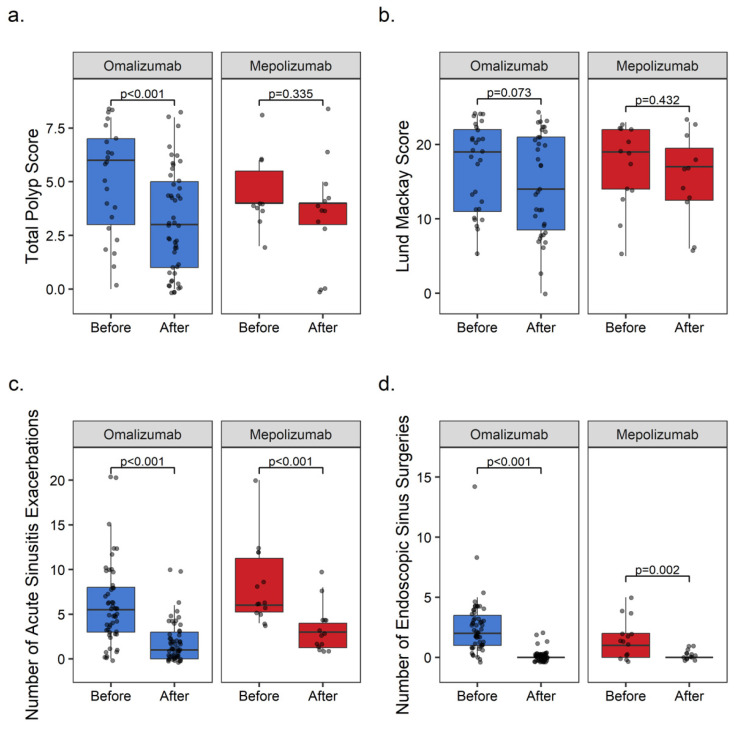
(**a**) Changes in endoscopic total nasal polyp scores, (**b**) changes in Lund–Mackay computed tomography scores, (**c**) decrease in acute exacerbations of chronic sinusitis, and (**d**) decrease in requirement of endoscopic sinus surgery due to nasal polyposis.

The post-treatment TPS were screened on the last visit of the patients. The median (IQR) TPS evaluation intervals were 35 (4–113) months for omalizumab and 13.5 (6–32) months for mepolizumab, as equal to the treatment duration. The only significant improvement in the TPS was observed with omalizumab. The median TPS score reduced with omalizumab from 6 [95% CI: 3–7] to 3 [95% CI: 2–4] (n = 23/55, *p* < 0.01). In the mepolizumab group, the median TPS was stable before and after treatment, being from 4 [95% CI: 3–6] to 4 [95% CI: 0–5] (n = 10/14, *p* = 0.335) (Figure 4a).

### 3.4. Lund–Mackay Computed Tomography Scores

No significant improvement in LMS was observed after treatment with either omalizumab or mepolizumab. The paranasal sinus opacities in the paranasal tomography findings of the patients after treatment were similar to the baseline findings in both of the BA groups. The median pre- and post-treatment LMS were 19 [95% CI: 12–21] vs. 14 [95% CI: 10–20] (n = 29/55, *p* = 0.073) in the omalizumab group, and 19 [95% CI: 13–22] vs. 17 [95% CI: 6–23] (n = 11/14, *p* = 0.432) in the mepolizumab group, respectively (Figure 4b). The median (min–max) pre-treatment and post-treatment CT scan interval of 36 (52.1%) patients with comparative CT was 36 months (6–130).

### 3.5. Acute Exacerbations of Chronic Rhinosinusitis

The secondary endpoints of our study were also to show a reduction in the frequency of acute exacerbation of chronic rhinosinusitis and the requirement of endoscopic sinus surgery due to nasal polyposis or chronic rhinosinusitis. A significant decrease in the frequency of acute exacerbation of chronic rhinosinusitis was observed in the patients with asthma-comorbid CRSwNP with the use of both of the BAs. It was shown that the median annual frequency of acute sinusitis exacerbations (AERCS) requiring antibiotics or systemic steroids was significantly reduced from 5.5 [95% CI: 4–6] to 1 [95% CI: 1–2] (*p* < 0.001) with omalizumab, and from 6 [95% CI: 5–12] to 3 [95% CI: 1–4] (*p* < 0.001) with mepolizumab (Figure 4c).

### 3.6. Endoscopic Sinus Surgery

Satisfactory results were obtained with biological treatment in the requirement of endoscopic sinus surgery (ESS), which impairs the patient’s quality of life and results in frequent relapses. The median total ESS frequency of patients, due to nasal polyposis, decreased significantly from 2 [95% CI: 2–3] to 0 [95% CI: 0–0] (*p* < 0.001) in the omalizumab group, and from 1 [95% CI: 0–4] to 0 [95% CI: 0–1] (*p* = 0.002) in the mepolizumab group (Figure 4d).

### 3.7. Advers Event

In the omalizumab group, only one patient (1.8%) described mild myalgia on the lower extremities after injection for 6 h. No adverse events were reported in the mepolizumab group [27].

## 4. Discussion

Here, we have designed a retrospective, observational, multicentric study based on the lack of national data on CRSwNP treatments and follow-up. For the first time across Türkiye, we have shown the efficacy of omalizumab and mepolizumab equally in CRSwNP treatment by real-life data.

We included 55 patients receiving omalizumab and 14 patients receiving mepolizumab in this study. Significant improvements were observed in patient-reported sinonasal outcomes, such as VAS, SNOT-22, and NCS, with both omalizumab and mepolizumab similarly.

TPSs were improved significantly after omalizumab treatment, but not after mepolizumab treatment. This could be explained by the limited patient population in the mepolizumab group.

In addition, we did not observe significant differences in LMS with both BAs.

For the BA-receiving patients diagnosed with CRSwNP, these scoring tools could be useful in monitoring the symptoms, however, these results should be evaluated together with endoscopic examinations and paranasal CT, as they may not always correlate with the severity of objective sinusitis findings and polyp dimensions. Reducing the paranasal CT opacities and endoscopic polyp sizes may take longer than the improvement in symptom scores.

Our study showed that, via omalizumab and mepolizumab treatments, the frequency of ESS and AECRS requiring systemic steroids or antibiotics was significantly reduced.

The BAs are promising agents for recurrent patients who have an inadequate response to standard medical treatments. They have shown a significant reduction in nasal polyp sizes and have increased the quality of life (QoL) of the CRSwNP patients with and without asthma [14,18].

BAs such as anti-IgE, anti-IL5, and anti-IL-4/IL-13 monoclonal antibodies (mAb) were found to be effective in the CRSwNP treatment of patients with severe asthma, with the most significant reduction being observed in the anti-IL-4R-mAb-receiving group [28].

The randomized double-blind, placebo-controlled phase three trials showed that omalizumab, mepolizumab, benralizumab, and dupilumab were all safe and effective in CRSwNP treatment. Both omalizumab and mepolizumab provided significant improvements in both symptom scores and TPS. The patients receiving dupilumab could not be included in this study. Although there are very few head-to-head comparative studies, dupilumab has been shown to be the agent with the highest effectiveness in CRSwNP treatment among biological agents [22,29,30,31].

Despite medical treatments, recurrent ESS is required in CRSwNP patients, which significantly reduces the QoL [8,11,32].

NERD (nonsteroidal anti-inflammatory drug (NSAID)-exacerbated respiratory disease) and pollen and mold sensitivity were similar in both groups, and house dust mite sensitivity was significantly higher in the omalizumab group (*p* < 0.001). The basal eosinophil count was significantly higher in the mepolizumab group (*p* = 0.003). Seven (17.7%) patients in the omalizumab group and two (14.3%) patients in the mepolizumab group had mold sensitivity (*p* = 1.000). In some of these cases, there were dens and destructive opacities suggestive of allergic fungal sinusitis in the paranasal sinuses. Due to the small number of patients in this subgroup, we could not perform their statistical analysis, but, interestingly, there were more extensive and resistant CT findings even after BA administration. 

The clinical trials of omalizumab, dupilumab, and mepolizumab, whose efficacy was demonstrated in CRSwNP treatment, did not include AFRS patients. Only a few published case reports and small studies have shown that these BAs provide a good response in the subgroup of AFRS patients [33,34,35,36]. This has aroused interest in further studies focusing specifically on the efficacy of BAs in AFRS patients.

As the indications for BAs have expanded, an increasing number of patients have begun to receive different combinations of BAs for their severe asthma or comorbid diseases. In the literature, there are a few reports showing the efficacy of omalizumab and mepolizumab dual treatment for asthma [37].

In this series, we had a 62-year-old female case with severe asthma, CRSwNP, and chronic spontaneous urticaria (CSU). While she was taking omalizumab with house dust sensitivity, she was switched to mepolizumab, due to the frequency of eosinophilic asthma attacks. Her asthma was well-controlled with mepolizumab, but her nasal polyps and CSU relapsed. For this reason, omalizumab was added to mepolizumab, and, at the end of 6 months of combination therapy, a reduction in polyp size was observed and CSU was well-controlled. 

There is a need to elucidate the additional genetic, epigenetic, and environmental factors that change the clinical course in patients with mixed endotypes that do not comply with the ‘one airway, one disease’ theory [38].

Türkiye, with the advantage of bridging Europe and Asia and receiving significant immigration, can enrich the exposure to various allergens and innate immunity in specific individuals [39].

This multicenter study is the most comprehensive real-life study evaluating the effects of both omalizumab and mepolizumab on CRSwNP in the Turkish population.

Since it is a retrospective cross-sectional study, the main limitations of our study are the heterogeneous number of patients, follow-up, and evaluation periods. Due to the small number of patients in the mepolizumab group, no comparison could be made between the two drug groups. The additional medical treatments, such as intranasal steroids, could not be presented, due to lack of medical records. 

Because of those limitations, in addition to the non-matching age, gender, and number of the two groups, a comparison of omalizumab and mepolizumab could not be made.

## 5. Conclusions

In conclusion, BAs are effective, safe, and promising treatments for challenging CRSwNP types. BA therapy selected by the endotype and phenotype may change the course of CRSwNP. Clinical and molecular biomarkers are needed to select the most appropriate candidate for biologics. Prospective real-life studies are needed to provide data on the comparative efficacy of BAs.

## Figures and Tables

**Table 1:** Baseline characteristics of the study population.

	Omalizumab (Anti-IgE)	Mepolizumab (Anti-IL-5)	Total	*p*-Value
Number of patients	55	14	69	
Age, median (IQR) years	43 (21–69)	43.5 (26–59)	43 (21–69)	0.720 ^2^
Female, n (%)	31 (56.4)	9 (64.3)	40 (58.0)	0.764 ^1^
Asthma duration, year, median (IQR)	13 (1–40)	14 (4–30)	13 (1–40)	0.840 ^2^
Asthma severity n, %				1.000 ^1^
Mild	2 (3.6)	0	2 (2.9)	
Moderate	5 (9.1)	1 (7.1)	6 (8.7)	
Severe	48 (87.3)	13 (92.9)	61 (88.4)	
Nasal polyposis duration, year, median (IQR)	11 (0–51)	14 (1–45)	12 (0–51)	0.846 ^2^
Total endoscopic sinus surgery, median (IQR)	2 (0–14)	1 (0–5)	2 (0–14)	0.120 ^2^
Omalizumab duration, month, median (IQR)	35 (4–113)			
Mepolizumab duration, month, mean (SD)		13.5 (6–32)		
Family history of nasal polyposis, n (%)	20 (36.4)	3 (21.4)	23 (33.3)	0.356 ^1^
Family history of asthma, n (%)	29 (52.7)	7 (50.0)	36 (52.2)	1.000 ^1^
Prick sensitization, n (%)				
Pollen	22 (40.0)	2 (14.3)	24 (34.8)	0.115 ^1^
House dust mite	47 (85.5)	2 (14.3)	49 (71.0)	<0.001 ^1^
Mold	7 (12.7)	2 (14.3)	9 (13.0)	1.000 ^1^
NERD, n (%)	35 (63.6%)	8 (57.1%)	43 (62.3%)	0.760 ^1^
Baseline eosinophil count, cells/µL, median (IQR)	630 (0–3800)	1175 (406–2400)	772 (0–3800)	0.003 ^2^
Baseline neutrophil count, cells/µL median (IQR)	4800 (2330–10,260)	4500 (2840–6530)	4600 (2330–10,260)	0.279 ^2^
Baseline total IgE, kU/L, median (IQR)	291 (21–2333)	280.5 (44–738)	291 (21–2333)	0.536 ^2^

IgE, immunoglobulin E; IL-5, interleukin-5; NERD, Nonsteroidal anti-inflammatory drug (NSAID)-exacerbated respiratory disease; SD, standard deviation. ^1^ Fisher’s Exact Test for Count Data. ^2^ Wilcoxon rank sum test.

## Data Availability

Data and materials are available upon request from the corresponding authors.

## Abbreviations

AECRS	acute exacerbations of chronic rhinosinusitis
AFRS	allergic fungal rhinosinusitis
BAs	biological agents
CI	confidence interval
CRS	chronic rhinosinusitis
CRSwNP	chronic rhinosinusitis with nasal polyps
CRSsNP	chronic rhinosinusitis without nasal polyps
eCRS	eosinophilic chronic rhinosinusitis
neCRS	non-eosinophilic chronic rhinosinusitis
CSU	chronic spontaneous urticaria
CT	computed tomography
ESS	endoscopic sinus surgery
GINA	Global Initiative for Asthma
IgE	immunoglobulin E
IL-4	interleukin-4
IL-4R	interleukin-4 receptor
IL-5	interleukin-5
LMS	Lund–Mackay score
mAb	monoclonal antibodies
MD	median difference
NCS	nasal congestion score
QoL	quality of life
PRO	patient-reported outcomes
SD	standard deviation
SNOT-22	sinonasal outcome test-22
TPS	endoscopic total nasal polyp scores
VAS	visual analogue scale
